# Spatial modeling of vaccine deserts as barriers to controlling SARS-CoV-2

**DOI:** 10.1038/s43856-022-00183-8

**Published:** 2022-11-10

**Authors:** Benjamin Rader, Christina M. Astley, Kara Sewalk, Paul L. Delamater, Kathryn Cordiano, Laura Wronski, Jessica Malaty Rivera, Kai Hallberg, Megan F. Pera, Jonathan Cantor, Christopher M. Whaley, Dena M. Bravata, Leslie Lee, Anita Patel, John S. Brownstein

**Affiliations:** 1 Computational Epidemiology Lab, Boston Children’s Hospital, Boston, MA USA; 2 Department of Epidemiology, Boston University School of Public Health, Boston, MA USA; 3 Harvard Medical School, Harvard University, Boston, MA USA; 4 Division of Endocrinology, Boston Children’s Hospital, Boston, MA USA; 5 Broad Institute of Harvard and MIT, Cambridge, MA USA; 6 Department of Geography and Carolina Population Center, University of North Carolina at Chapel Hill, Chapel Hill, NC USA; 7 SurveyMonkey, San Mateo, CA USA; 8 The COVID Tracking Project at the Atlantic, District of Columbia, USA; 9 Castlight Health, San Francisco, CA USA; 10 RAND Corporation, Santa Monica, CA USA; 11 Center for Primary Care and Outcomes Research, Stanford University School of Medicine, Stanford, CA USA; 12 National Center for Immunization and Respiratory Diseases, Centers for Disease Control and Prevention, Atlanta, GA USA

**Keywords:** Health services, Public health, Epidemiology, Disease prevention, Infectious diseases

## Abstract

**Background:**

COVID-19 vaccine distribution is at risk of further propagating the inequities of COVID-19, which in the United States (US) has disproportionately impacted the elderly, people of color, and the medically vulnerable. We sought to measure if the disparities seen in the geographic distribution of other COVID-19 healthcare resources were also present during the initial rollout of the COVID-19 vaccine.

**Methods:**

Using a comprehensive COVID-19 vaccine database (VaccineFinder), we built an empirically parameterized spatial model of access to essential resources that incorporated vaccine supply, time-willing-to-travel for vaccination, and previous vaccination across the US. We then identified vaccine deserts—US Census tracts with localized, geographic barriers to vaccine-associated herd immunity. We link our model results with Census data and two high-resolution surveys to understand the distribution and determinates of spatially accessibility to the COVID-19 vaccine.

**Results:**

We find that in early 2021, vaccine deserts were home to over 30 million people, >10% of the US population. Vaccine deserts were concentrated in rural locations and communities with a higher percentage of medically vulnerable populations. We also find that in locations of similar urbanicity, early vaccination distribution disadvantaged neighborhoods with more people of color and older aged residents.

**Conclusion:**

Given sufficient vaccine supply, data-driven vaccine distribution to vaccine deserts may improve immunization rates and help control COVID-19.

## Plain language summary

COVID-19 has affected the elderly, people of color, and individuals with chronic illnesses more than the general population. Large barriers to accessing the COVID-19 vaccine could make this problem worse. We used a website called VaccineFinder, which has information on the location of most COVID-19 vaccine doses in the US, to measure vaccine accessibility in early 2021. We then identified vaccine deserts, defined as small US regions with poor access to the COVID-19 vaccine. We found that over 10% of the US lived in a vaccine desert. Overall, we found that vaccines were less available to people in rural areas, people of color, and individuals with chronic illnesses. It will be important to reverse this pattern and ensure enough vaccines are sent to these communities to help reduce the spread of COVID-19.

## Abstract (web summary)

Rader et al. model spatial accessibility to COVID-19 vaccines in the US, identifying vaccine deserts where access was insufficient during the first phase of the rollout. Rural areas and medically vulnerable populations are found to have poorer access to vaccines, as are areas with higher proportions of Black, Hispanic and older residents.

## Introduction

Geographic access to medical resources is an essential component to maximizing the benefits of healthcare^1^. Individuals who must travel longer to access services are less likely to receive routine, acute, and pandemic-related care^2–4^. To achieve an equitable distribution of resources, spatial barriers should be minimized for all communities and access to care should be ensured for those who need it the most^1^. This is especially true for the distribution of the COVID-19 vaccine in the United States (US) given the notable disparities in the virus’s impact^5^. A disproportionate burden of COVID-19 morbidity and mortality has been experienced among individuals with a lower median income and Black and Hispanic persons^6–8^. These differences are associated with significant inequities in the ability to physically distance^9,10^, burden of co-morbidities^7,11^, percent of people in essential worker roles^7,9,12^, and systemic disparities in the likelihood to die without receiving care^13^. Further, reduced geographic access to SARS-CoV-2 testing in rural areas, areas with a higher percentage of non-White persons, and areas with a large percentage of uninsured individuals suggests that the disproportionate impact of the virus is likely understated^14^.

The introduction of multiple vaccines creates the potential to reduce the burden of COVID-19 in the US^15^. Mitigating disparities and ensuring a fair and equitable distribution of the COVID-19 vaccine are guiding principles of the Centers for Disease Control and Prevention (CDC) distribution recommendations^16^. However, an ideal distribution of the vaccine may be difficult to achieve because it relies on current medical supply chains and distribution locations, which are efficient but demographically biased^14^. The need to vaccinate large numbers of individuals at a highly elevated risk for COVID-19 (e.g., healthcare workers and long-term care residents and staff)^17^ led to initial allocation in most states being focused on hospitals and academic medical centers with supply later being expanded to other clinics and pharmacies. However, these facilities have been shown to be unequally distributed throughout the US and less geographically accessible to rural populations^18–21^. Reliance on these nodes for distribution of the COVID-19 vaccine can lead to vaccine deserts, where local supply is not sufficiently accessible or available.

Studies in Chicago, Baltimore and Los Angeles have shown a higher prevalence of low-access pharmacy deserts in minority communities and neighborhoods with higher rates of infectious disease^19,22,23^ while a state-wide study in Pennsylvania showed that pharmacy deserts tend to be in rural communities^20^. Scenario modeling suggests these pharmacy disparities may result in unequal distribution of the COVID-19 vaccine^24^, but to our knowledge no studies have measured geographic accessibility to confirmed vaccine locations or attempted to account for differences in vaccine supply. In addition, traditional definitions of resource deserts (e.g., food within 10 miles^25^), are insufficient to characterize the unique goals of rapid, population-wide immunization. To quantify accessibility of the COVID-19 vaccine, we utilize a unique comprehensive dataset of COVID-19 vaccine providers in the US and employ the enhanced two-step floating catchment area (E2SFCA) method^18^, a technique that has been used to study accessibility to various health resources including primary care physicians^18^, pharmacies^26^, and COVID-19 healthcare^27^. This flexible spatial model simultaneously incorporates supply, demand, travel time, and between-census-tract travel.

In this analysis, we seek to understand if COVID-19 vaccine doses were optimally allocated to unvaccinated individuals in communities that are most vulnerable to the virus during the early vaccine rollout. To accomplish this, vaccine distribution sites and vaccine supply quantities in the contiguous US states were identified in early 2021 from a comprehensive national database (VaccineFinder). Utilizing the E2SFCA method, we calculated a spatial accessibility score for each US Census tract, which was the outcome of interest. To connect the relationship of spatial accessibility to disease transmission, we define vaccine deserts with a novel modification of resource desert that comports with COVID-19 specific public health goals. Utilizing US Census demographic data and a high-resolution survey of 16.5 million Facebook users^28–30^, we find that early vaccine distribution resulted in vaccine deserts in rural locations and communities with medically vulnerable populations. To identify if disparities in spatial accessibility to the COVID-19 vaccine exist within communities of similar urbanicity, we apply spatial regression modeling. Our regression findings suggest early vaccination disadvantaged neighborhoods with more Black, Hispanic, and older aged residents. To understand this further, we explore a case-series of two highly racially segregated US cities: Detroit, Michigan and Chicago, Illinois. Lastly, we model potential vaccine deserts under conditions where supply is no longer constrained to understand relative contributions of supply shortages to vaccine deserts.

## Methods

### Demographic and population data

We gathered demographic and cartographic data from the US Census 2015-2019 American Community Survey^31^ for each census tract in the contiguous US with a non-zero population count (*n* = 72,042, Alaska and Hawaii excluded due to the unique geographic challenges). We calculated population density (people per square kilometer) and the proportional breakdowns by race, sex, median resident age, median income, health insurance type, and internet access. We gathered information about urbanicity (e.g., metropolitan, rural) from the US Department of Agriculture Rural-Urban Commuting Area (RUCA) codes^32^ and population counts from the Gridded Population of the World (v4) dataset^33^. Vaccination rates on February 16, 2021 for each US county were obtained from the National Center for Immunization and Respiratory Diseases^34^.

### COVID-19 and medical burden data

We estimated the COVID-19 and medical burden for each census tract using data from the Delphi Group at Carnegie Mellon University U.S. COVID-19 Trends and Impact Survey (*N* = 16,533,319) conducted via the Facebook platform (April 6, 2020–February 11, 2021)^28,29^. This survey was chosen because its large sample size allowed for exploring fine spatial resolutions. Participants in this survey were recruited from clicking a banner on their Facebook newsfeed. The survey was hosted on Qualtrics and adults 18 years and older were surveyed after providing explicit informed consent^30^.

Medical burden was defined as the self-reported proportion of respondents with at least one pre-existing condition that could exacerbate COVID-19 severity (Supplementary Table). COVID-19 burden was defined as the proportion of surveys responding “yes” to the question “in the past 24 h, have you had direct contact with anyone who recently tested positive for COVID-19 (coronavirus)” for each week, which closely mirror government case counts in the US^35,36^, although the latter are only available at county-level resolutions (Supplementary Fig. 1).

### COVID-19 vaccine locations and supply data

The location and supply of publicly accessible COVID-19 vaccine administration sites in the contiguous US were gathered from VaccineFinder, a comprehensive, national vaccine system maintained by Boston Children’s Hospital in collaboration with the CDC and Castlight Health. VaccineFinder receives registration information and daily reports of on-hand vaccine supply for each COVID-19 vaccine administration site from the CDC’s Vaccine Tracking System (VTrckS), the ordering platform for entities receiving COVID-19 vaccines^37^. VTrckS is the most comprehensive vaccine supply source because all 64 public health jurisdictions and pharmacies participating in the FRPP and receiving direct allocation from the federal government are enrolled^38^. All vaccine distribution sites are collected in the database, however each location can choose to display their location, vaccine availability, and/or appointment resources to the public on the web-based search tool (www.VaccineFinder.org).

We used all vaccine distribution sites (*N* = 37,287) that administered one or more COVID-19 vaccine dose on at least three separate days between February 17, 2021–March 16, 2021 (doses administered was imputed from supply levels, Supplementary Methods under dose administration days). The overall dose count for each site was determined by calculating the mean daily doses administered following each site’s receipt of its first reported dose. U.S. Department of Veteran Affairs (VA) locations (extracted from their publicly available database^39^) dispensing the vaccine were also included (*N* = 139). The supply levels for VA vaccine distribution sites were determined by the daily reported difference (February 19, 2021–March 17, 2021) in cumulative doses administered as reported on February 20, 2021.

### Spatial accessibility outcome

We calculated spatial accessibility of vaccine supply using the E2SFCA^18^. Scores were undefined for a small number (*n* = 23) of census tracts, resulting in estimates for the majority (*n* = 72,019) of the populated tracts in the contiguous US. The first step of E2SFCA calculates the vaccine supply-to-population ratio (*R*_*j*_) for each vaccine distribution site (*j*) based on the mean daily supply (*S*_*j*_) of a site and the unvaccinated population (*P*) of its catchment area (*k* ∈ {*d*_*kj*_ ≤ T}) (Supplementary Fig. 2, Step 1). A catchment area is defined as the set of all grid squares (*k*) where the distance (*d*) between that grid square and the vaccine distribution site is less than or equal to a threshold, T. Daily supply levels were multiplied by 248 to represent the total doses over the aspirational roll out period (January 1–September 6), assuming the current modeled supply was constant over time. September 6 (Labor Day) was chosen to coincide with the expiration of the majority of COVID-19 related unemployment benefits provided by the American Rescue Plan Act of 2021^40^. These assumptions were chosen to reflect circumstances at the time of analysis. The model used here has the flexibility to incorporate alternative assumptions in the presence of expected change (increases in vaccine supply, etc.).

The catchment populations were calculated using unvaccinated population counts and weighted by the travel-time decay function (*W*_*d*_) based on willingness to travel to receive the vaccine (see below for more details). Unvaccinated population counts and locations were calculated from the 1 km^2^ resolution gridded data^33,34^. The population count for each grid square was adjusted to only include the percentage unvaccinated by multiplying the grid total by the percentage unvaccinated (from the county the grid square is in) plus ½ the percentage partially vaccinated. For computational efficiency, we set T equal to 90 min.

1 $${R}_{j}=\frac{{S}_{j}* 248}{{\sum }_{k\in \left\{{d}_{{kj}}\le {{{{{\rm{T}}}}}}\right\}}{P}_{k}{W}_{d}}$$

The second step of the E2SFCA (Supplementary Fig. 2, Step 2) calculated the spatial accessibility value (*A*) for each census tract (*i*) by summing all the travel-time-weighted supply-to-population ratios (*R*_*j*_ *W*_*d*_) for each vaccine distribution site within its own catchment area. Each census tract is represented by its population-weighted centroid^41^. As in the first step, the catchment area is delineated by a travel threshold around each census tract and the supply-to-population ratio is weighted by the travel-time decay function.

2 $${A}_{i}={\sum }_{j\in \left\{{d}_{{ij}}\le {{{{{\rm{T}}}}}}\right\}}{R}_{j}{W}_{d}$$

### Travel time

Travel time (*d*) was determined by utilizing an impedance map of the quickest transversal time (e.g., car, public transportation, boat, etc.) across the gridded population surface of the US^42–44^. For each vaccine distribution location, travel time from all grid squares within 90 min were measured by calculating the lowest accumulated travel time across a friction surface between the vaccine location and the grid square. This calculation was made using Dijkstra’s algorithm^45^ which calculates the fastest total travel time between a source and destination point by tracing the fastest travel time between each pair of neighboring intervening points and optimizing the fastest cumulative path across these pairs. The same procedure was used to determine the catchment area for each census tract by measuring the travel time between each grid square and population-weighted centroid of the census track.

### Time willing to travel to vaccine and travel-time decay function

**Fig. 1:**
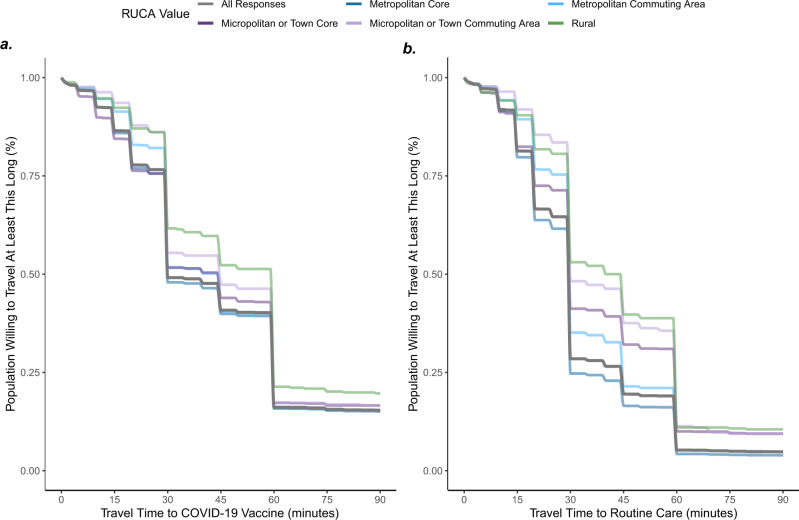
Time willing to travel to COVID-19 vaccine compared to routine healthcare. Responses (*N* = 23,288) from OutbreaksNearMe US national web survey on how long people were willing to travel to COVID-19 vaccine (**a**) compared to routine care (**b**). Lines are monotonic cubic splines fit to 1 minus the empirical cumulative distribution of these response times. Responses were limited to individuals considering a vaccine, defined as those who reported either “yes” or “not sure” when asked if they would get the vaccine. Results are broken down by U.S. Department of Agriculture Rural-Urban Commuting Area (RUCA) codes of the respondent’s self-reported zip code. Overall distribution of travel time to COVID-19 vaccine (**a** *gray*) was used as the travel-time decay function in E2SFCA model.

To empirically define the time unvaccinated individuals would be willing to travel for the COVID-19 vaccine, we included travel time questions within a large-scale, nationally-representative, previously-validated^46^ web survey from OutbreaksNearMe (ONM) via the SurveyMonkey platform (survey fielded February 9–February 17, 2021, see reference^47^ for full survey language and Supplementary Table 2 for survey demographics). The proportion of respondents previously vaccinated for COVID-19 was calculated using survey weights. We then asked all unvaccinated survey participants: “What’s the most time you would be willing to travel to receive a COVID-19 vaccine? (please enter a time in minutes).” To ensure comparability to other health resources, we also asked “What’s the most time you would be willing to travel for a routine medical appointment? (please enter a time in minutes).” Responses were limited to 1–90 min (T in the spatial accessibility model). We received 23,288 responses among those (*N* = 26,466, 88.0%) presented the question. Travel times from survey respondents who indicated they were considering COVID-19 vaccination (e.g., responded “yes” or “not sure” when asked if they would get the vaccine) were used to define our travel-time decay function^48,49^ (*W*, Fig. 1, left, plotted as gray line) via a monotonic cubic spline fit to 1 minus the empirical cumulative distribution of these responses. The resulting spline^50^ approximates a continuous travel-time delay function (such as the commonly used Gaussian function^51^) but captures the different behavioral response to time benchmarks (e.g., perceived difference between 59 min and 61 min).

### Definition of vaccine desert

Access deserts for essential resources (e.g., food, pharmacy) are typically defined in terms of a resource lacking within a specific travel-time or distance^19,23,25^. However, these definitions are restrictive given the lack of consideration of potential supply and our empirical analysis of the long times individuals will travel for a vaccine. Therefore, we used the E2SFCA accessibility score to incorporate supply and travel time into the vaccine distribution scheme model: assuming the vaccine sites available to each census tract have a constant supply for the 8-month period (January–September 6, 2021), each tract would have a travel-time weighted sum of *A*_*i*_ doses per person in their respective weighted catchment areas. Access strata therefore broadly represent cutoffs suggesting difficulty in reaching herd immunity (modeled at 85% based on variants circulating at time of analysis, assumptions detailed in Supplementary Methods under herd immunity estimates) in 8-months with one dose (vaccine desert, *A*_*i*_ < 0.85), immunizing all with one dose (low, 0.85 ≤ *A*_*i*_ < 1.0), difficulty immunizing all with two doses (medium, 1.0 ≤ *A*_*i*_ < 2.0), and sufficient accessibility (high, *A*_*i*_ ≥ 2.0). Note these thresholds are based on assumptions of the reproductive number, vaccine effectiveness, desired time to achieve herd immunity, a constant supply, and vaccines available and variants circulating at the time of analysis. Though a simplification, the model allows flexibility to relax these assumptions as additional data become available or if other public health priorities are selected. We have conducted a sensitivity analysis varying the accessibility score cutoff to 0.75 (based on lower herd immunity) and 0.95 (based on higher herd immunity) to assess this assumption.

### Spatial regression model

Based on the known distribution of health resources^52^, we hypothesized that vaccine distribution would be a mixture of consolidated vaccine sites (e.g., medical centers) and intentionally spaced locations to capture markets (e.g., pharmacies, rural health clinics). This may create spatial autocorrelation and spillover such that the location of one vaccine distribution site (and resulting accessibility scores) may be dependent on the placement of sites around them. The E2SFCA also allows individuals to access sites across census boundaries, potentially amplifying correlation in accessibility scores when census tracts rely on overlapping distribution points. To quantify the determinants of accessibility accounting for autocorrelation of accessibility scores in neighboring census tracts and urban/rural confounding, we fit a spatial lag model:^53,54^

3 $${A}_{i} = {g}{QA}_{i} + {\beta }_{1}{log}({Population} \; {Density}_{i}) + {\beta}_{2}{{RUCA}}_{i} + {\beta}_{3}{X}_{i} + {\varepsilon}$$

where the term *g QA*_*i*_ represents the coefficient (*g*) and spatially lagged standardized accessibility score (*QA*_*i*_) from a weighted matrix (queen contiguity) of neighboring census tracts. Sensitivity analyses of k-nearest (*k* = 4) neighbor inverse distance weighted matrix was performed. We modeled the log of population density and multiple other predictor variables (*X*) for each census tract (*i*). The models include six basic multivariate models (urbanicity measured by RUCA code and population density in addition to a single demographic or disease burden metric) and one full multivariate model (all modeled variables detailed in Table 2). We then used Monte Carlo simulation to quantify the resulting direct impact^55^, to produce a coefficient similar to Ordinary Least Squares regression and irrespective of spillover effects to neighboring census tracts. Census tracts with incomplete demographic data (*N* = 390, 0.5%) and outlier (see Supplementary Methods under census tract outliers) accessibility scores (*N* = 1699, 2.3%) were excluded from regression analysis.

### Flooded supply simulation

To understand vaccine deserts that may persist when vaccine distribution is abundant, we re-run our E2SFCA catchment area model setting the supply-to-population ratio (*R*_*j*_) to 2. This implies all sites have enough vaccine to theoretically serve every person in their catchment area.

Calculations were performed in R version 3.6.2. The ONM survey was approved by the Boston Children’s Hospital Institutional Review Board (IRB-P00023700) and received a waiver of informed consent. The Delphi Group survey protocol required informed consent, collected via a Qualtrics survey platform. It was approved by the Carnegie Mellon University IRB (STUDY2020_00000162). This activity was reviewed by CDC and was conducted consistent with applicable federal law and CDC policy.^§^

^§^See e.g., 45 C.F.R. part 46, 21 C.F.R. part 56; 42 U.S.C.

^§^241(d); 5 U.S.C.

^§^552a; 44 U.S.C.

^§^3501 et seq.

### Reporting summary

Further information on research design is available in the Nature Research Reporting Summary linked to this article.

## Results

### Time willing to travel among those considering vaccination

**Fig. 2:**
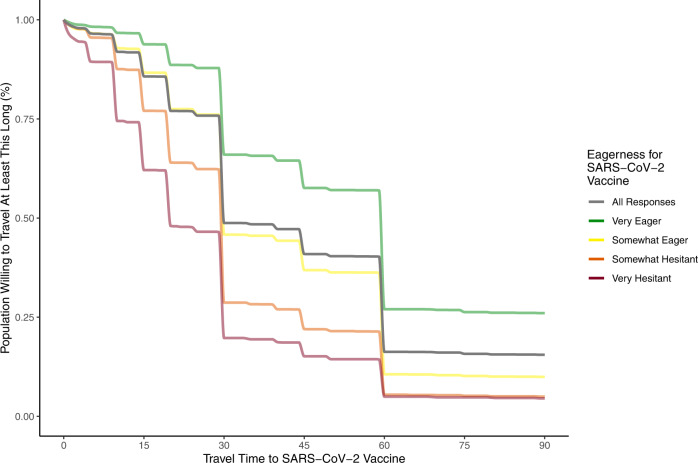
Population willingness to travel to COVID-19 vaccine by vaccine hesitancy. Responses (*N* = 23,288) to OutbreaksNearMe U.S. national web survey on how long people were willing to travel for the COVID-19 vaccine stratified by self-reported vaccine hesitancy. Lines are monotonic cubic splines fit to 1 minus the empirical cumulative distribution of these response times. Responses were limited to individuals considering a vaccine, defined as those who reported either “yes” or “not sure” when asked if they would get the vaccine.

In the ONM survey^47^, 19% of survey respondents reported having received at least one dose of COVID-19 vaccine. The majority of ONM survey respondents (81%) were unvaccinated and, of these, most (*N* = 23,288) indicated “yes” (61.2%) or “not sure” (21.2%) when asked if they wanted the COVID-19 vaccine. These individuals considering the vaccine indicated they were willing to travel a median of 44.0 [IQR: 30.0–60.0] minutes to receive a COVID-19 vaccine (Fig. 1a) versus 33.9 [IQR: 20–45] to receive routine medical care (Fig. 1b). Respondents were on average willing to travel 10.6 min (paired two-sided *t*-test 95% CI: 10.2–10.9) longer to get a vaccine compared to routine care. Notably, while there was a substantial gap (12.1 min, two-sided *t*-test 95% CI: 9.9–14.3) between time willing to travel to routine care along an urban-rural divide (31.9 min for individuals in urban zip codes versus 44.0 min mean time in rural zip codes) this difference was reduced (6.8 min, two sided *t*-test 95% CI: 4.3–9.3) for travel to the COVID-19 vaccine. Willingness to travel was more strongly related to vaccine hesitancy (Fig. 2) than the urban-rural divide, with individuals who were “very eager” to get the vaccine willing to travel a mean of 27.2 min longer (95% CI: 25.9–28.5, *p* < 0.001) than those who were “very hesitant.”

### Geographic distribution of vaccine deserts

**Fig. 3:**
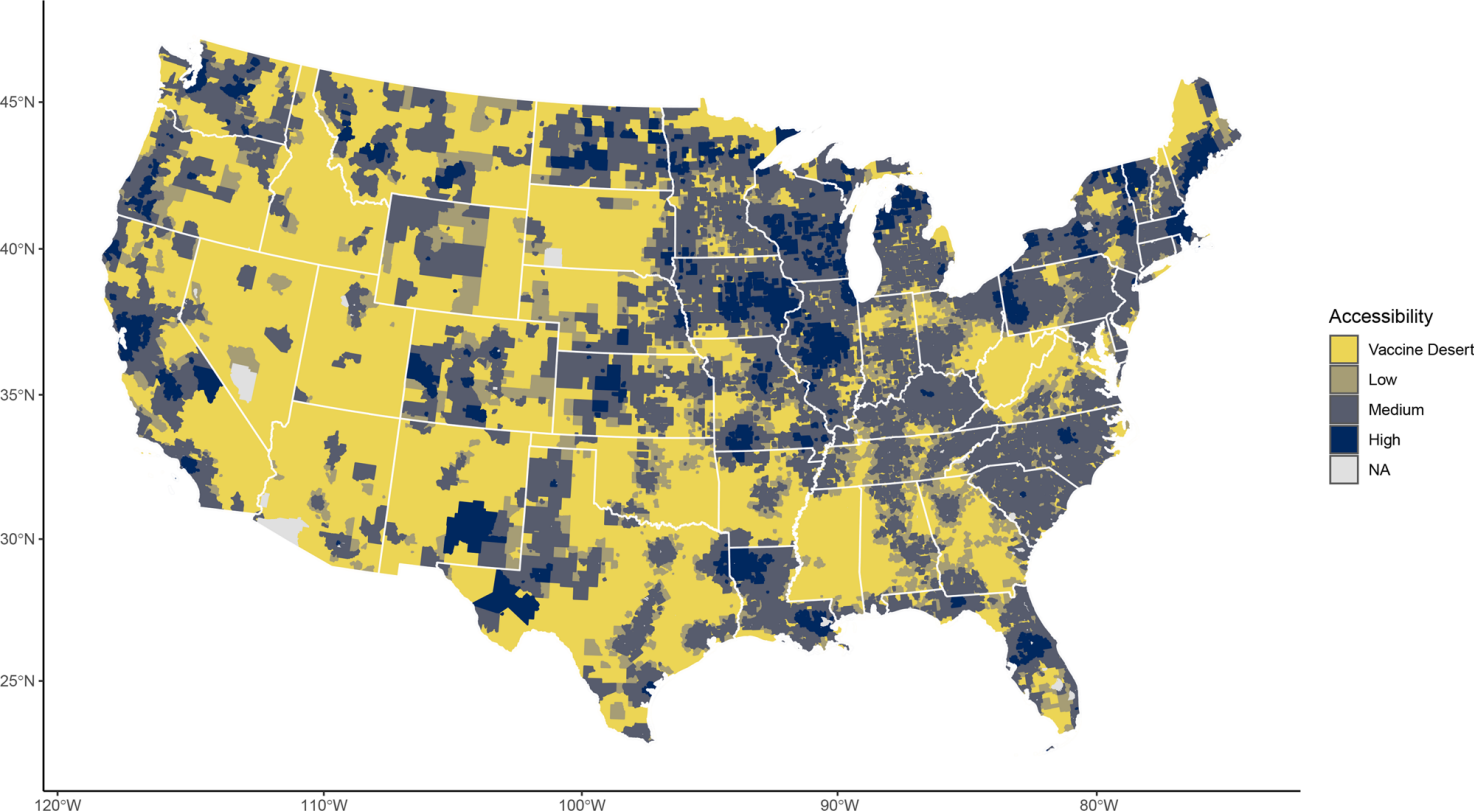
COVID-19 vaccine deserts and accessibility scores across the US. Map of United States Census tracts colored by their spatial accessibility to COVID-19 vaccine doses. Accessibility scores (*A*_*i*_) were estimated by the enhanced two-step floating catchment area method. Access strata broadly represent cutoffs suggesting difficulty in reaching herd immunity in 8-months (Jan 1—American Labor Day) with one dose (vaccine desert, *A*_*i*_ < 0.85), immunizing all with one dose (low, 0.85 ≤ *A*_*i*_ < 1.0), difficulty immunizing all with two doses (medium, 1.0 ≤ *A*_*i*_ < 2.0), and sufficient accessibility (high, *A*_*i*_ ≥ 2.0). Under the modeled distribution scheme, if the vaccine sites available to each census tract have a steady supply for the rollout period, they would have a sum (accounting for travel-time decay) of *A*_*i*_ doses per person in their respective weighted catchment areas. No estimates (NA) were provided for non-populated census tracts and those (*n* = 23) where *A*_*i*_ was undefined.

Using the self-reported time-willing-to-travel distribution to parametrize an E2SFCA model of accessibility to the COVID-19 vaccine sites, we found considerable geographic disparities of vaccine access across the US (Fig. 3**)**. There was good accessibility to the vaccine in the Pacific region where over 93% of census tracts were categorized as medium or high accessibility (Table 1). Access was also found to be strong in New England and the Middle Atlantic where respectively only 3.2% and 4.1% of census tracts were categorized as vaccine deserts (Table 1). Conversely, 27.8% of tracts in the Mountain region were categorized as vaccine deserts as well as 18.9% in West- and 22.4% in East–South Central regions.

**Table 1:** Census tract demographics by accessibility score for the Contiguous United States

	Vaccine desert	Low accessibility	Medium accessibility	High accessibility	All
	N Tracts: 7999	N Tracts: 4582	N Tracts: 45,283	N Tracts: 714,155	N Tracts: 72,019
	Column percent	Column percent	Column percent	Column percent	Row percent
**Total population (*N*, %)**	32,847,516 (10.2%)	20,899,652 (6.5%)	205,655,647 (63.8%)	63,009,659 (19.5%)	322,412,474 (100.0%)
**Census division (*N*, %)**					
New England	109 (3.2%)	215 (6.4%)	1552 (46.2%)	1486 (44.2%)	3362 (4.7%)
Middle Atlantic	408 (4.1%)	323 (3.2%)	7757 (77.0%)	1582 (15.7%)	10,070 (14.0%)
East North Central	711 (6.1%)	662 (5.7%)	7361 (62.8%)	2979 (25.4%)	11,713 (16.3%)
West North Central	523 (9.9%)	272 (5.2%)	3244 (61.6%)	1231 (23.4%)	5270 (7.3%)
South Atlantic	1743 (12.9%)	1148 (8.5%)	9668 (71.3%)	998 (7.4%)	13,557 (18.8%)
East South Central	994 (22.4%)	614 (13.8%)	2782 (62.7%)	46 (1.0%)	4436 (6.2%)
West South Central	1533 (18.9%)	682 (8.4%)	5040 (62.2%)	844 (10.4%)	8099 (11.2%)
Mountain	1448 (27.8%)	499 (9.6%)	2751 (52.7%)	520 (10.0%)	5218 (7.2%)
Pacific*	530 (5.1%)	167 (1.6%)	5128 (49.8%)	4469 (43.4%)	10,294 (14.3%)
**Rural-Urban designation (*N*, %)**					
Metropolitan Core	1856 (3.6%)	2022 (3.9%)	35,186 (68.2%)	12,559 (24.3%)	51,623 (71.7%)
Micropolitan/Town Core	1468 (23.3%)	772 (12.3%)	3359 (53.3%)	700 (11.1%)	6299 (8.7%)
Metropolitan Commuting Area	1832 (24.7%)	941 (12.7%)	4103 (55.2%)	556 (7.5%)	7432 (10.3%)
Micropolitan/Town Com. Area	1472 (41.8%)	485 (13.8%)	1388 (39.4%)	177 (5.0%)	3522 (4.9%)
Rural	1371 (43.6%)	362 (11.5%)	1247 (39.7%)	163 (5.2%)	3143 (4.4%)
**Female (mean %, SD)**	49.8 (±5.1)	50.4 (±4.7)	50.9 (±4.8)	50.9 (±4.3)	50.8 (±4.8)
Missing (*N*, %)	16 (0.2%)	8 (0.2%)	84 (0.2%)	22 (0.2%)	130 (0.2%)
**Median age (mean years, SD)**	42.7 (±8.2)	40.9 (±8.3)	39.4 (±7.8)	38.1 (±7.0)	39.6 (±7.8)
Missing (*N*, %)	16 (0.2%)	9 (0.2%)	102 (0.2%)	24 (0.2%)	151 (0.2%)
**Median income (mean $1,000, SD)**	28.5 (±9.2)	30.8 (±10.7)	33.5 (±13.8)	35.7 (±16.3)	33.2 (±13.9)
Missing (*N*, %)	27 (0.3%)	12 (0.3%)	142 (0.3%)	32 (0.2%)	213 (0.3%)
**Race/ethnicity (mean %, SD)**					
White	80.4 (±20.8)	79.8 (±20.9)	72.0 (±25.2)	66.4 (±26.2)	72.3 (±25.1)
Black	9.7 (±16.9)	11.5 (±18.9)	15.2 (±22.6)	13.2 (±22.2)	13.9 (±21.8)
Hispanic	11.8 (±17.3)	11.2 (±15.9)	17.1 (±21.7)	20.1 (±23.7)	16.7 (±21.5)
American Indian or Alaskan Native*	2.6 (±10.7)	1.2 (±4.8)	0.7 (±2.9)	0.6 (±2.3)	0.9 (±4.6)
Asian	1.6 (±3.6)	2.0 (±3.5)	4.4 (±7.7)	9.0 (±13.4)	4.9 (±8.9)
Hawaiian or Pacific Islander*	0.1 (±0.5)	0.1 (±0.4)	0.1 (±0.5)	0.2 (±0.8)	0.1 (±0.6)
Two or more races	2.7 (±2.8)	2.9 (±2.7)	3.1 (±2.6)	3.7 (±3.2)	3.1 (±2.8)
Other race	2.9 (±6.5)	2.4 (±4.5)	4.6 (±8.4)	6.9 (±11.1)	4.7 (±8.7)
Missing (*N*, %)	16 (0.2%)	8 (0.2%)	84 (0.2%)	22 (0.2%)	130 (0.2%)
**No internet access (mean %, SD)**	20.7 (±11.5)	16.6 (±10.3)	14.6 (±10.3)	13.6 (±9.7)	15.2 (±10.6)
Missing (*N*, %)	30 (0.4%)	23 (0.5%)	206 (0.5%)	45 (0.3%)	304 (0.4%)
**Insurance coverage (mean %, SD)**					
Employer	39.9 (±12.6)	43.6 (±13.7)	45.4 (±15.1)	47.6 (±15.4)	45.1 (±14.9)
Direct	6.4 (±4.1)	6.3 (±3.9)	6.6 (±4.2)	6.6 (±4.3)	6.5 (±4.2)
Medicare	6.8 (±3.4)	6.0 (±3.4)	5.4 (±3.2)	4.8 (±2.6)	5.5 (±3.2)
Medicaid	15.2 (±9.5)	14.2 (±9.5)	15.7 (±12.2)	17.3 (±13.4)	15.8 (±12.1)
Veterans Administration	0.4 (±0.5)	0.3 (±0.5)	0.3 (±0.5)	0.2 (±0.5)	0.3 (±0.5)
Tricare	1.1 (±3.8)	1.5 (±5.1)	1.0 (±4.0)	0.5 (±2.4)	0.9 (±3.8)
Two or More	6.4 (±3.3)	6.4 (±3.2)	6.0 (±3.3)	5.7 (±3.1)	6.0 (±3.2)
Uninsured	10.4 (±6.6)	9.5 (±6.8)	9.0 (±7.3)	7.3 (±6.0)	8.8 (±7.0)
Missing (*N*, %)	25 (0.3%)	20 (0.4%)	154 (0.3%)	31 (0.2%)	230 (0.3%)
**Medical burden**^**†**^ **(mean %, SD)**	53.5 (±5.9)	52.5 (±5.4)	49.0 (±6.2)	46.5 (±6.7)	49.2 (±6.5)
Missing (*N*, %)	63 (0.8%)	11 (0.2%)	79 (0.2%)	14 (0.1%)	167 (0.2%)
**COVID-19 burden**^**†**^ **(mean %, SD)**	3.1 (±1.4)	3.0 (±1.1)	2.6 (±1.1)	2.4 (±1.1)	2.7 (±1.1)
Missing (*N*, %)	63 (0.8%)	11 (0.2%)	79 (0.2%)	14 (0.1%)	167 (0.2%)

*Alaska and Hawaii excluded from analysis.

^†^Percentage of survey respondents in each census tract who reported COVID-19 related pre-existing conditions (medical burden) or previous contact with COVID-19 positive individual (COVID-19 burden).

### Vaccine deserts in relation to demography

Vaccine deserts are home to 10.2% (*N* = 32,847,516) of the US census estimated population. A sensitivity analysis varying the accessibility score cutoff for vaccine desert to 0.75 and 0.95, found that 21,289,659 (6.6%) and 45,385,859 (14.1%) people would live in a desert, respectively. When stratifying census tracts by RUCA code, spatial accessibility is higher in metropolitan cores (92.5% medium or high accessibility). This falls dramatically with RUCA classifications farther from metropolitan cores, dropping from 64.4% (micropolitan/town core) to 44.9% (rural area). Rural and micropolitan/town commuting areas had the worst accessibility; vaccine deserts comprised 43.6% and 41.8% of census tracts in these areas, respectively.

In contrast to other forms of vaccine access, disparities in spatial access largely reflect the demographics of the urban-rural divide. Vaccine deserts as compared to high accessibility locations are concentrated among older (4.5 year more), white (14.0% more), lower income ($7190 less), and uninsured (3.1% higher) census tract populations. Vaccine deserts also have a greater burden of those with COVID-19-associated medical conditions (6.9% higher), COVID-19 exposure (0.7% higher), and less access to the internet (7.1% less). These relationships scale monotonically, and low and medium accessibility areas have differences that generally fall linearly between vaccine deserts and high accessibility locations.

### Determinants of spatial accessibility

To understand the determinants of accessibility scores accounting for urban-rural confounding, we performed a multivariate analysis and separate spatial regression models for each variable of interest while controlling for population density and urbanicity. While a simplification, a census tract’s accessibility score can be generally interpreted as the number of COVID-19 doses accessible to each unvaccinated individual living in it over the 248-day study period. According to a direct impact assessment from the multivariate model (best fitting according to Akaike information criterion), census tracts with the highest quartile of Black and Hispanic residents were associated with 0.024 and 0.016 lower accessibility scores respectively, when compared to the lowest quartile for each variable (Supplementary Data 1). Per 100,000 individuals in census tracts with the highest quartile of Black and Hispanic residents, this broadly translates to 2400 and 1600 fewerless accessible COVID-19 vaccine doses over the study period. Census tracts with older residents were associated with 200 fewer accessible doses per 100,000 people for each additional year of age, while census tracts with a higher prior COVID-19 burden were associated with 49,700 fewer accessible doses per 100,000 people for each additional percent of previous infection. We find the opposite effect for medical burden whereby, when controlling for population density, an increased percentage of individuals with self-reported pre-existing conditions was associated with 8300 more accessible doses per 100,000 people over the study period. Direction of results were robust to a sensitivity analysis with an alternative weighting matrix (Supplementary Data 2).

### Case study of racial disparities in highly segregated urban centers

**Fig. 4:**
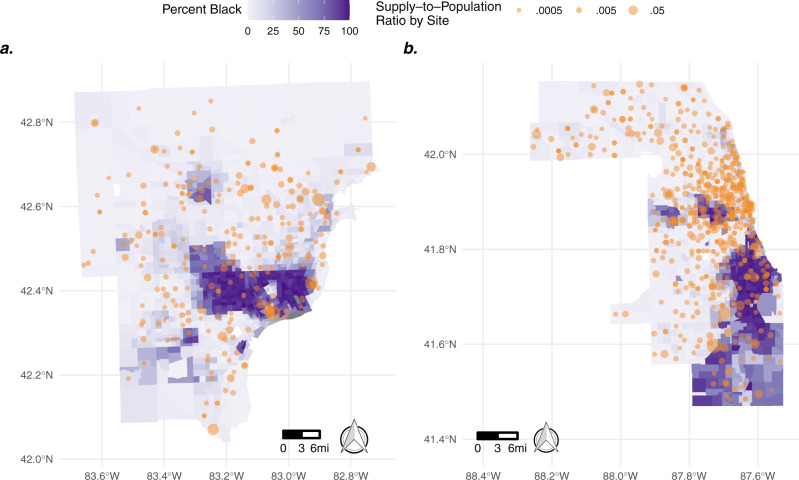
Disparities in COVID-19 vaccine access in Detroit and Chicago, USA. Map of census tracts around Detroit, Michigan (**a**) and Chicago, Illinois (**b**) by the percentage of residents who are Black (*purple*). Overlayed in orange are COVID-19 vaccine distribution locations sized by their supply-to-population ratio. Vaccine distribution sites (from Vaccines.gov) in both cities are sparser in census tracts with a higher percentage of Black residents.

To explore differential access by race within areas of the similar urban composition, we focused on two highly racially segregated cities, Detroit (defined here: Wayne, Oakland, and Macomb Counties, MI) and Chicago (Cook County, IL)^56^. Consistent with our findings of high vaccine accessibility scores in metropolitan cores, neither of these cities have census tracts that meet the vaccine desert criteria, and most tracts have numerically high accessibility to the COVID-19 vaccine (*Mean A*_*i*_ = 1.8 in Detroit and 2.2 in Chicago). Despite this, we find notable racial disparities in the organization of vaccine distribution in both locations. We find census tracts with a higher proportion of Black residents were served by noticeably fewer vaccine distribution sites compared to nearby tracts with fewer black residents in both Detroit (38% less, Fig. 4a) and Chicago (28% less, Fig. 4b). Of note, vaccine distribution sites located in Black neighborhoods in Detroit were more centralized consisting of fewer locations with larger supply, while tracts with a larger percentage of non-Black residents were served by a distributed network of smaller sites. Numerically, there were 406 more daily doses available per 100,000 residents in majority (>50%) Black census tracks, despite these tracts being served by less vaccine distribution locations. Unlike in Detroit, in Chicago we found that the largest distribution sites were located in predominately non-Black tracts of the city. Quantitatively, this translated to majority Black census tracts in Chicago having 120 less daily doses available per 100,000 residents during the study period.

### Flooded supply simulation

When repeating our model under the assumption each vaccine distribution site has enough vaccine doses for every individual in its catchment area, we find that only 106 US census tracts persist as vaccine deserts (Supplementary Fig. 3) which is 1.3% of vaccine deserts under the present constrained supply conditions. These locations are primarily located in the rural Mountain and Pacific divisions of the US and the majority of them are outside or on the outskirts of the modeled 90-min distribution site catchment areas as evidenced by accessibility scores at or near zero (Supplementary Fig. 4).

## Discussion

The distribution of the COVID-19 vaccine supply during the timeframe of February 19, 2021–March 17, 2021 has created vaccine deserts that have the potential to interfere with population-wide disease control. Based on an empirically parameterized spatial accessibility model, paired with an extensive national dataset of confirmed locations with vaccines and their doses administered, we find that the initial distribution scheme favored spatial accessibility within US metropolitan areas. In contrast, vaccine deserts, more often found in rural regions, had a preponderance of vulnerable populations. Vaccine deserts had more residents with self-reported COVID-19 exposures and pre-existing conditions as well as more individuals who lacked health insurance. When measuring determinants of accessibility while controlling for population density, we see that tracts with a higher proportion of Black residents, Hispanic residents and older residents had reduced spatial access to the vaccine. Together, these findings highlight that at the time of our analysis (February 19–March 17 2021) vaccine dissemination points within the US were not yet effectively placed to target those most vulnerable to COVID-19.

US populations living in rural census tracts had the lowest accessibility to the COVID-19 vaccine. While prior studies hypothesize that rural populations may be willing to travel further distances for health resources than those in urban areas^57,58^ we find these differences were diminished for the COVID-19 vaccine. Despite a large portion of urban populations’ willingness to travel longer for the vaccine than they would for routine care, this effect was reduced for those in rural census tracts. This finding may be related to rural populations reporting higher levels of vaccine hesitancy^59^, which our findings show had a stronger association with willingness to travel than geography. These regions may benefit the most from the COVID-19 vaccine due to their relatively higher medical burden^60^, older population^61^ and lower likelihood to consider adoption of mitigation measures such as face mask wearing^46^. We also find that convenience could play a large role in whether hesitant individuals would be willing to get vaccinated, suggesting increased spatial access may be a meaningful public health intervention. Only ~50% of the overall population reported willingness to travel >30 min for the vaccine and <20% would travel >1 h. These numbers were even smaller amongst hesitant individuals. Despite rural areas being less populated than urban ones, after incorporating these findings, our model suggests ~14% of the US lived in a vaccine desert at the time of analysis. Inability to reach spatially coupled rural regions with vaccinations presents the risk of intense outbreaks^62^ and may sustain persistent chains of transmission which are barriers to infection elimination^63^, although the latter has yet to be studied for SARS-CoV-2.

COVID-19 morbidity and mortality is not distributed equally across demographic groups and prior work has shown that within both urban^9,13^ and rural^64^ regions, communities with a higher proportion of minorities had worse outcomes. Ensuring equal access to the COVID-19 vaccine is an important part of mitigating these structural racism outcomes and preventing further propagation of these imbalances. Previous state-wide analyses have shown that pharmacy deserts are more likely to be in rural regions with a high white population^20^, while citywide analyses have shown they tend to be located in neighborhoods with more minorities^19,22,23^. Utilizing a 90-min catchment area to understand vaccine deserts (the definition of which is specific to COVID-19 immunization goals, but more flexible than the strict radius limits routinely used to study access to less scarce resources^19,23^), our results are congruent with both these findings. On the national level, we observe vaccine deserts in rural census tracts that were predominately white. Yet, when we remove urban-rural confounding and look within regions of similar spatial structure, we find the same racial disparities highlighted in the pharmacy desert literature, suggesting vaccine distribution strategies during this timeframe did not go far enough to address these disparities.

When adjusting for urban-rural confounding we find higher spatial accessibility for those with self-reported pre-existing conditions, which may highlight deliberate vaccine site placement or the likelihood of individuals who live near medical resources to receive diagnoses^65^. While self-reports are biased estimates of medical diagnoses, this also suggests that our general finding of higher burden in rural areas may be an underestimate given a rural region’s reduced access to diagnoses. We also find that, despite the initial targeting of older individuals for vaccination, census tracks with a higher median age had less spatial accessibility during the study period. This highlights how a concerted effort needs to be made to overcome demographic biases in infrastructure and optimally allocate medical resources to their intended target.

Importantly, the geospatial barriers discussed here only represent one type of vaccine access challenge. Social disparities (e.g., ability to take time off work, obtain child care, procure efficient transportation) suggest equal spatial barriers can manifest very differently^66,67^ and that vulnerable populations may be disproportionately affected by poor spatial access. For example, this is supported by evidence suggesting that individuals with lower income experienced a disproportionate reduction in access to health services in response to the pandemic^68^. The two-dose regimens for the selected COVID-19 vaccine formulations available at the time of analysis also created a need to access a vaccine distribution site twice within a short period (21 or 28 days), suggesting that spatial barriers may compound and/or affect dose completion rates. Additionally, the disparities in internet access in vaccine deserts suggest these regions may have had a harder time both identifying and accessing vaccine distribution locations due to the reliance on web-based tools (e.g., Vaccines.gov) to find vaccine availability and schedule appointments. These same disparities in internet access may affect the representativeness of the two surveys used here and cause undersampling in vaccine deserts (which census data shows have poorer internet access). Therefore, our results may underestimate the medical and COVID-19 burden in census tracts with poor spatial accessibility to the COVID-19 vaccine. Panel surveys are also only sampling individuals willing to complete them. For questions regarding vaccine intent, this group may not be representative of the broader US or hold true over time, as perceptions relating to vaccination can change quickly^69^.

The VaccineFinder dataset used in this analysis is likely the most comprehensive source of vaccine site and dose supply information given the mandate for COVID-19 vaccine providers enrolled in the Federal Retail Pharmacy Partnership (FRPP) to register in VTrckS. However, not all locations modeled may have been open or advertising to the public and we only included locations with evidence of dose administration on multiple occasions. These locations will likely change substantially as vaccine supply expands to more providers. Additionally, while we integrate locations from the Veterans Administration, our dataset is missing sites in states that only partially joined FRPP (e.g., West Virginia) and other important federal vaccine distribution channels such as Military Treatment Facilities, the Indian Health Service and Federally Qualified Health Centers. The missing sites are therefore likely not distributed at random which may bias the association between demographics and vaccine accessibility in either direction.

The current analysis also only measured spatial access from the perspective of the unvaccinated dose recipient (and assumed everyone currently unvaccinated in the population was eligible to receive a vaccine) based on static observations of vaccination locations in the early phases of the rollout process and the time-willing-to-travel of those who had yet to vaccinate (which ignores the long distances people may have been willing to travel in the first phase of the rollout). Distribution during this period was characterized by manufacturing constraints^17^ and supply-chain barriers (e.g., need for ultralow temperature freezers)^70^, as well as prioritized vaccination of healthcare workers, those over 65 years of age, and certain high-risk individuals^17^. The selected site locations during the time of this analysis favor urban areas to optimally reach these populations for vaccination. Populations with a high number of medical workers may be vaccinated relatively quickly, allowing some distribution nodes to broaden their population served. Conversely, populations with a high number of children under 18 have a large proportion of residents who may not yet be eligible for the vaccine. Additionally, this analysis did not account for natural immunity levels already in the population which is rapidly changing and may not be evenly geographically distributed. Here we have shown the importance of changes in the vaccine desert cutoff based on herd immunity, highlighting the sensitivity of this assumption.

Improving the supply of vaccines will cause the numerator of the vaccine supply-to-population ratio to rise and minimize the number of vaccine deserts as defined here. When scenario modeling a situation in which supply is abundant, we find that very few vaccine deserts remain suggesting that increases in supply will help alleviate the presently modeled deserts. However, as we remove supply barriers, travel time to vaccine sites will become the primary component of accessibility. These travel time metrics have been shown to have similar demographic disparities as the present results when measuring accessibility of other COVID-19 resources^14,71,72^. Further, even in locations such as Detroit and Chicago, where vaccine supply and spatial accessibility is numerically adequate (i.e., not a desert), locations of vaccine distribution sites may favor certain groups and exacerbate disparities. Coordinated efforts are required to ensure distribution is both data-driven and optimized for local circumstances.

As vaccine priorities evolve, states increase/shift distribution locations and supply, and one-dose vaccines are integrated into the supply-chain, the barriers identified here can be overcome and the results from this study period will change. This manuscript presents a novel utilization of a highly flexible model that can incorporate these future changes while also helping identify the need to direct vaccine supply to underserved communities. Controlling COVID-19 in the US requires extensive, well-orchestrated coordination of vaccine delivery at the national and local scales^73^. Here we show significant disparities in geographic access to the COVID-19 vaccine during the early stages of the vaccination program, a hurdle to this achievement. Eliminating COVID-19 vaccine deserts and spatial barriers to transmission control will require an ongoing commitment to optimizing distribution to align with continued expansion of populations prioritized to receive vaccines and simultaneous investment in resources to mitigate transportation access disparities. This will necessitate locally targeted interventions including active outreach campaigns, mobile vaccination clinics and other innovative health delivery mechanisms such as vaxathons^74^ and ride-share partnerships^75^.

## Supplementary information

Reporting Summary

Description of Additional Supplementary Files

Supplementary Information

Supplementary Data 1

Supplementary Data 2

## Data Availability

Real-time vaccine site location is available at VaccineFinder.org for locations that choose to publicly display. Vaccine dose supply is available: [https://data.cdc.gov/Vaccinations/Vaccines-gov-COVID-19-vaccinating-provider-locatio/5jp2-pgaw]. Source data for manuscript main figures available: [10.6084/m9.figshare.20513949]. Delphi Group COVID-19 Trends and Impact Survey data may requested via [https://cmu-delphi.github.io/delphi-epidata/].

## Code availability

Code and travel-time decay function is available: github.com/computationalepi/E2SFCA and archived on Zenodo^50^.
