# Supplementary material for: Spatial modeling of vaccine deserts as barriers to controlling SARS-CoV-2
Source: Commun Med (Lond). 2022 Nov 10;2:141. doi: 10.1038/s43856-022-00183-8 (PMC9649755; doi:10.1038/s43856-022-00183-8)
Supplement: Supplementary file 1 — Reporting Summary [file 43856_2022_183_MOESM1_ESM.pdf]

nature research

Corresponding author(s): Brownstein
Last updated by author(s): Sep 2, 2022

Reporting Summary

Nature Research wishes to improve the reproducibility of the work that we publish. This form provides structure for consistency and transparency in reporting. For further information on Nature Research policies, see our Editorial Policies and the Editorial Policy Checklist.

Statistics

For all statistical analyses, confirm that the following items are present in the figure legend, table legend, main text, or Methods section.

n/a	Confirmed	
☐	☒	The exact sample size (n) for each experimental group/condition, given as a discrete number and unit of measurement
☐	☒	A statement on whether measurements were taken from distinct samples or whether the same sample was measured repeatedly
☐	☒	The statistical test(s) used AND whether they are one- or two-sided<br>*Only common tests should be described solely by name; describe more complex techniques in the Methods section.*
☐	☒	A description of all covariates tested
☐	☒	A description of any assumptions or corrections, such as tests of normality and adjustment for multiple comparisons
☐	☒	A full description of the statistical parameters including central tendency (e.g. means) or other basic estimates (e.g. regression coefficient) AND variation (e.g. standard deviation) or associated estimates of uncertainty (e.g. confidence intervals)
☐	☒	For null hypothesis testing, the test statistic (e.g. F, t, r) with confidence intervals, effect sizes, degrees of freedom and P value noted<br>*Give P values as exact values whenever suitable.*
☒	☐	For Bayesian analysis, information on the choice of priors and Markov chain Monte Carlo settings
☒	☐	For hierarchical and complex designs, identification of the appropriate level for tests and full reporting of outcomes
☒	☐	Estimates of effect sizes (e.g. Cohen's d, Pearson's r), indicating how they were calculated

Our web collection on statistics for biologists contains articles on many of the points above.

Software and code

Policy information about availability of computer code

Data collection	N/A
Data analysis	Code and travel-time decay function is available: github.com/computationalepi/E2SFCA and archived on Zenodo.

For manuscripts utilizing custom algorithms or software that are central to the research but not yet described in published literature, software must be made available to editors and reviewers. We strongly encourage code deposition in a community repository (e.g. GitHub). See the Nature Research guidelines for submitting code & software for further information.

Data

Policy information about availability of data

All manuscripts must include a data availability statement. This statement should provide the following information, where applicable:

- Accession codes, unique identifiers, or web links for publicly available datasets
- A list of figures that have associated raw data
- A description of any restrictions on data availability

Real-time vaccine site location is available at VaccineFinder.org for locations that choose to publicly display. Vaccine dose supply is available: [https://data.cdc.gov/Vaccinations/Vaccines-gov-COVID-19-vaccinating-provider-locatio/5jp2-pgaw]. Source data for manuscript main figures available: [doi.org/10.6084/m9.figshare.20513949]. Delphi Group COVID-19 Trends and Impact Survey data may requested via [https://cmu-delphi.github.io/delphi-epidata/].

Field-specific reporting

Please select the one below that is the best fit for your research. If you are not sure, read the appropriate sections before making your selection.

☐ Life sciences ☒ Behavioural & social sciences ☐ Ecological, evolutionary & environmental sciences

For a reference copy of the document with all sections, see nature.com/documents/nr-reporting-summary-flat.pdf

Behavioural & social sciences study design

All studies must disclose on these points even when the disclosure is negative.

Study description	Used the enhanced two-step floating catchment area method to measure spatial accessibility to SARS-CoV-2 vaccine locations in the United States.
Research sample	Outcome was spatial accessibility calculated on 22,806 SARS-CoV-2 vaccine distribution locations.
Sampling strategy	Multiple web survey instruments used. End-page river sampling was used for randomization.
Data collection	Survey data was collected via the web on the SurveyMonkey and Facebook platforms
Timing	February 19, 2021 and March 17, 2021
Data exclusions	N/A
Non-participation	Unknown
Randomization	N/A

Reporting for specific materials, systems and methods

We require information from authors about some types of materials, experimental systems and methods used in many studies. Here, indicate whether each material, system or method listed is relevant to your study. If you are not sure if a list item applies to your research, read the appropriate section before selecting a response.

Materials & experimental systems

n/a	Involved in the study	
☒	☐	Antibodies
☒	☐	Eukaryotic cell lines
☒	☐	Palaeontology and archaeology
☒	☐	Animals and other organisms
☐	☒	Human research participants
☒	☐	Clinical data
☒	☐	Dual use research of concern

Methods

n/a	Involved in the study	
☒	☐	ChIP-seq
☒	☐	Flow cytometry
☒	☐	MRI-based neuroimaging

Human research participants

Policy information about studies involving human research participants

Population characteristics	U.S. adults age 18 and older. See supplementary table 1 to exact for exact population characteristics.
Recruitment	Subjects were recruited on the surveymonkey and facebook platforms. Subjects were not financially or otherwise incentivized to participate.
Ethics oversight	The ONM survey was approved by the Boston Children's Hospital Institutional Review Board (IRB-P00023700) and received a waiver of informed consent. The Delphi Group survey protocol was approved by the Carnegie Mellon University IRB (STUDY2020_00000162). The Delphi survey was hosted on Qualtrics and individuals were surveyed after providing explicit informed consent.

Note that full information on the approval of the study protocol must also be provided in the manuscript.
