# Supplementary material for: Spatial modeling of vaccine deserts as barriers to controlling SARS-CoV-2
Source: Commun Med (Lond). 2022 Nov 10;2:141. doi: 10.1038/s43856-022-00183-8 (PMC9649755; doi:10.1038/s43856-022-00183-8)
Supplement: Supplementary file 2 — Description of Additional Supplementary Files [file 43856_2022_183_MOESM2_ESM.pdf]

Description of Additional Supplementary Files

File Name: Supplementary Data 1

Description: Spatial Regression on Accessibility to COVID-19 Vaccine Score (A_i) for each US Census Tract

File Name: Supplementary Data 2

Description: Spatial Lag Regression on Accessibility to COVID-19 Vaccine Score for each US Census Tract using k-Nearest Neighbor (k=4) Inverse Distance Weight Matrix
