# Supplementary material for: Spatial modeling of vaccine deserts as barriers to controlling SARS-CoV-2
Source: Commun Med (Lond). 2022 Nov 10;2:141. doi: 10.1038/s43856-022-00183-8 (PMC9649755; doi:10.1038/s43856-022-00183-8)
Supplement: Supplementary file 3 — Supplementary Information [file 43856_2022_183_MOESM3_ESM.pdf]

Supplementary Information
Spatial Modeling of Vaccine Deserts as Barriers to Controlling SARS-CoV-2

Supplementary Methods

Doses Administration Days

The VaccineFinder database contains the number of doses each site reported per day. Doses administered was measured as the difference between doses each day and the day prior. Days with negative values (e.g., more doses on Tuesday than Monday) were considered "supply days" and excluded from analysis. Only locations with three or more dose administration days were included. This method allowed for the systematic exclusion of centralized warehouses reporting large dose quantities, but not administering doses to individuals. This approach may lead to underestimating total dose quantity administered in exchange for better capturing precise locations where doses are being administered.

Zip to Census Tract Mapping and Validation

Zip codes were mapped to census tracts such that each individual survey response contributed to all census tracts it overlapped with, proportional to the percentage of overlap (as measured by residential addresses)[2,3]. For example, a survey response from zip 40025 contributed 0.99 persons to census tract 21111007502 and 0.01 persons to census tract 21111010318 because this zip code had a 99% and 1% overlap with the census tracts, respectively. Census tracts containing under 10.0 total persons were excluded from analyses.

The goal of this sub-analysis is to understand how accessibility relates to comorbidities and COVID-19 burden at the census tract level. However, some variables such as COVID-19 burden are only measured at the county and zip code level as opposed to the census tract. Of primary concern is whether zip code data mapped to census tract in the above-described method (hence "derived census tract") is an adequate representation of what is happening at the census tract level. An alternative is to use lower resolution county data as a stand-in for census tract variables.

Given that the average age of residents is available at all three spatial resolutions, we compare correlations in this known variable to understand which method may be best to represent variables unmeasured in census tracts (e.g., COVID burden). First, we compare percentage of survey respondents over age 65 in each derived census tract with ACS estimates of individuals over 65 in each census tract (Pearson's r = .65). Next, we compare the percentage of individuals over 65 in each census tract to the same variable inherited from the county estimate (Pearson's r = .49). While neither is a perfect representation of what is happening in the census tract, utilizing derived census tracts explains more variation in actual census tract age than using estimates inherited from a county resolution.

Case Study City Boundaries

Detroit was defined by Wayne, Oakland and Macomb County borders. Chicago was defined by Cook County borders.

Census Tracts Outliers

Outliers were census tracts with accessibility scores greater than 2 standard deviations over the mean. Qualitatively, outliers were generally remote census tracts, adequately served by a single vaccine location, with little competition for doses within 90 minutes.

Herd Immunity Estimates

$$V_{c_1} = \frac{1}{Ve_1} * (1 - \frac{1}{R_0})$$

One dose vaccine coverage (V_{c_1}) is estimated to be approximately 85% as a function of:

- The approximate mean first dose infection vaccine efficacy (Ve_1) of the BNT162b2 and mRNA-1273 (80%) vaccines following appropriate waiting period[4]
- A mean estimated basic reproductive number (R_0) of SARS-CoV-2 (3.28)[5]
- Assuming random mixing and persistent immunity
- We did not include pre-existing infection derived immunity

Supplementary Figure 1: Validation of Disease Burden Data from Delphi Survey

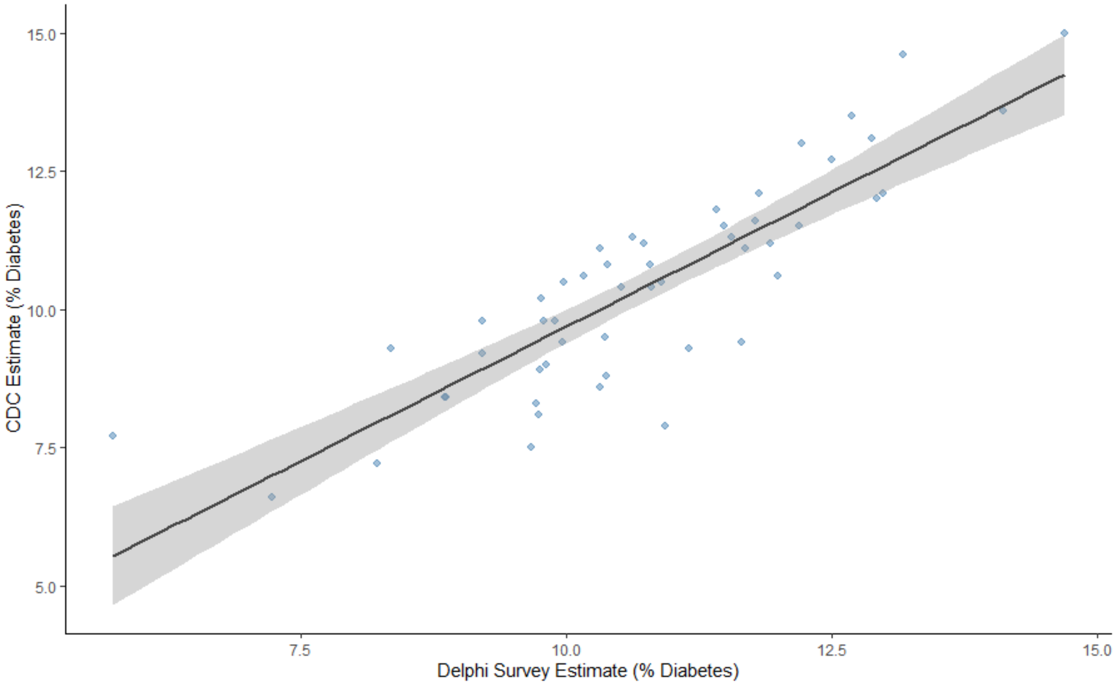

Estimates of diabetes from the US Delphi web survey compared to US Centers for Disease Control and Prevention estimates of adults diagnosed with diabetes[1] for each of the 50 states and the District of Columbia (Pearson's r = .86).

Supplementary Figure 2: Example of Step One and Two of Enhanced Two Step Catchment Area Method from Massachusetts and Surrounding States

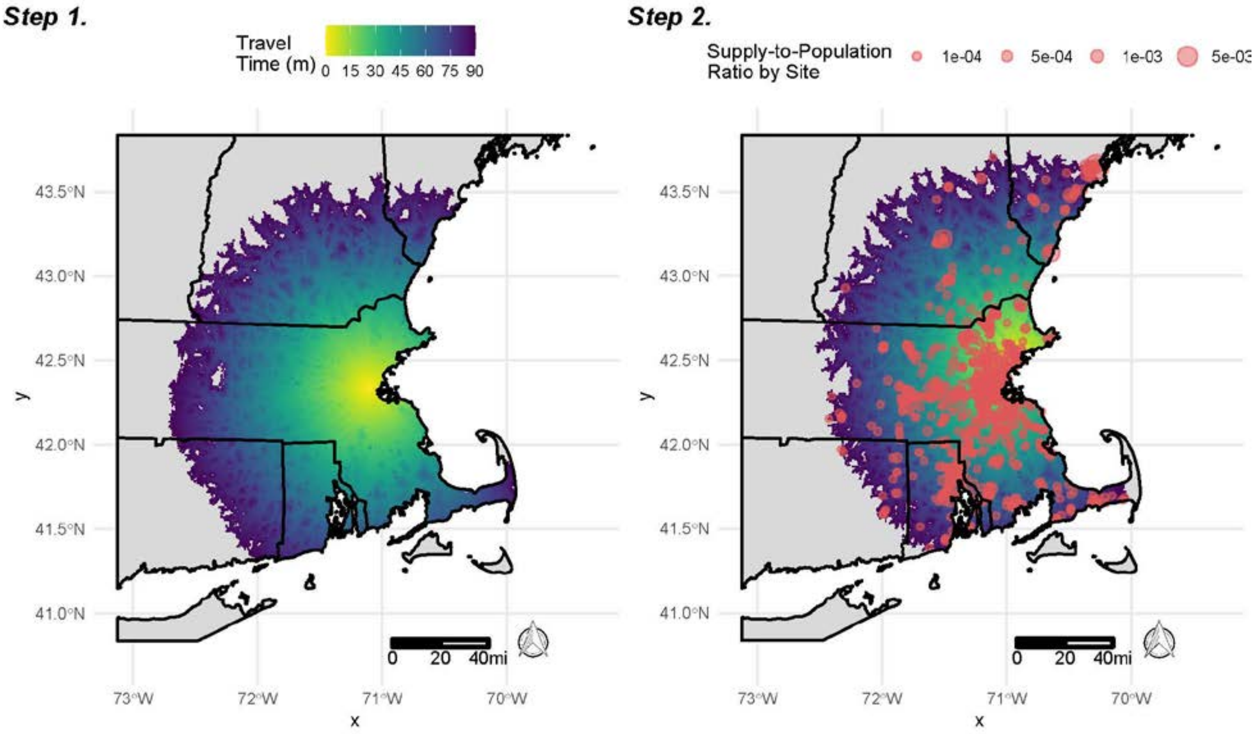

The first step of E2SFCA is to determine the supply-to-population ratio of each vaccine provider. The catchment area (the denominator of this equation) is defined as the population in each 1km grid square within 90 minutes of the site, weighted by the travel time from the grid square to the site (e.g., each site can serve 100% the population within 1 minute of the site and 50% of the population 40 minutes away). The numerator of the equation is determined by the daily doses on hand for each site. This process is repeated for each distribution location (*left*, shows example from Boston Children's Hospital Site). The second step is to calculate accessibility scores of each census tract by taking the sum of all the supply-to-population ratios (*salmon*) from within 90 minutes of the location. The supply-to-population ratios from sites further away are similarly downweighed. This step is repeated for each census tract in the United Sates (*right*, shows example from census tract in Salem, MA)

Supplementary Figure 3: COVID-19 Vaccine Deserts and Accessibility Scores Across the United States When Simulating Abundant Vaccine Supply

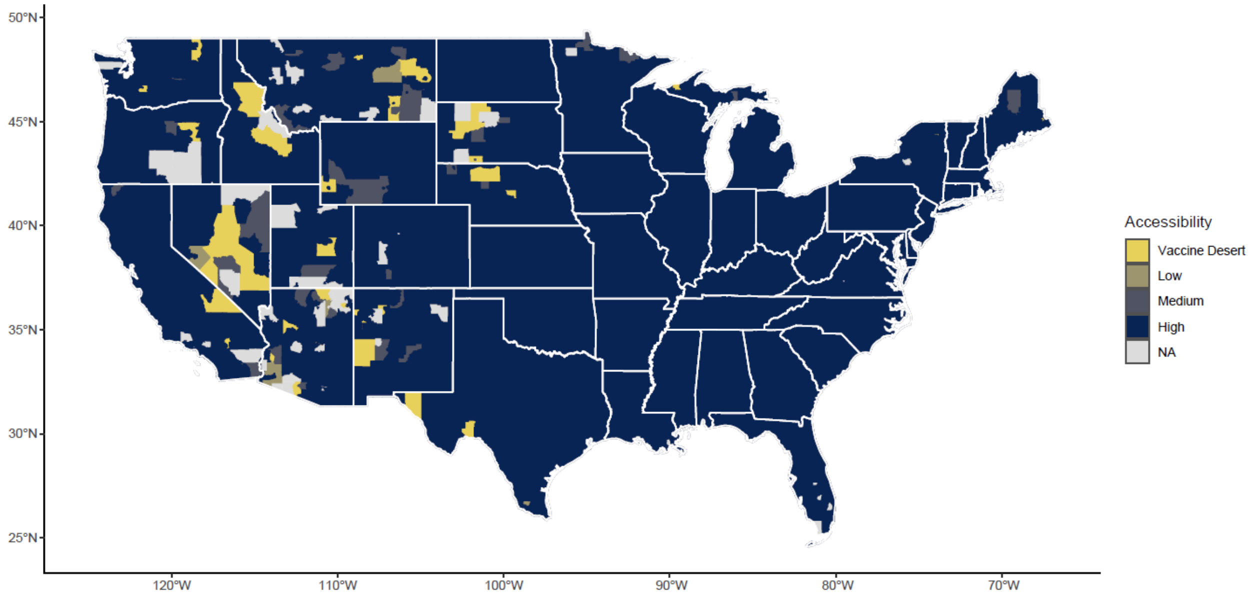

Map of United States Census tracts colored by their spatial accessibility to COVID-19 vaccine doses. Accessibility scores (A_i) were estimated by the enhanced two-step floating catchment area method under a condition of flooded vaccine supply (supply-to-population ratio set to 2 for all vaccine distribution locations). Access strata broadly represent cutoffs suggesting difficulty in reaching herd immunity in 8-months (Jan 1 – American Labor Day) with one dose (vaccine desert, $A_i < 0.85$) immunizing all with one dose (low, $0.85 \leq A_i < 1.0$), difficulty immunizing all with two doses (medium, $1.0 \leq A_i < 2.0$), and sufficient accessibility (high, $A_i \geq 2.0$).

Supplementary Figure 4: Histogram of Vaccine Desert Accessibility Scores When Simulating Abundant Vaccine Supply

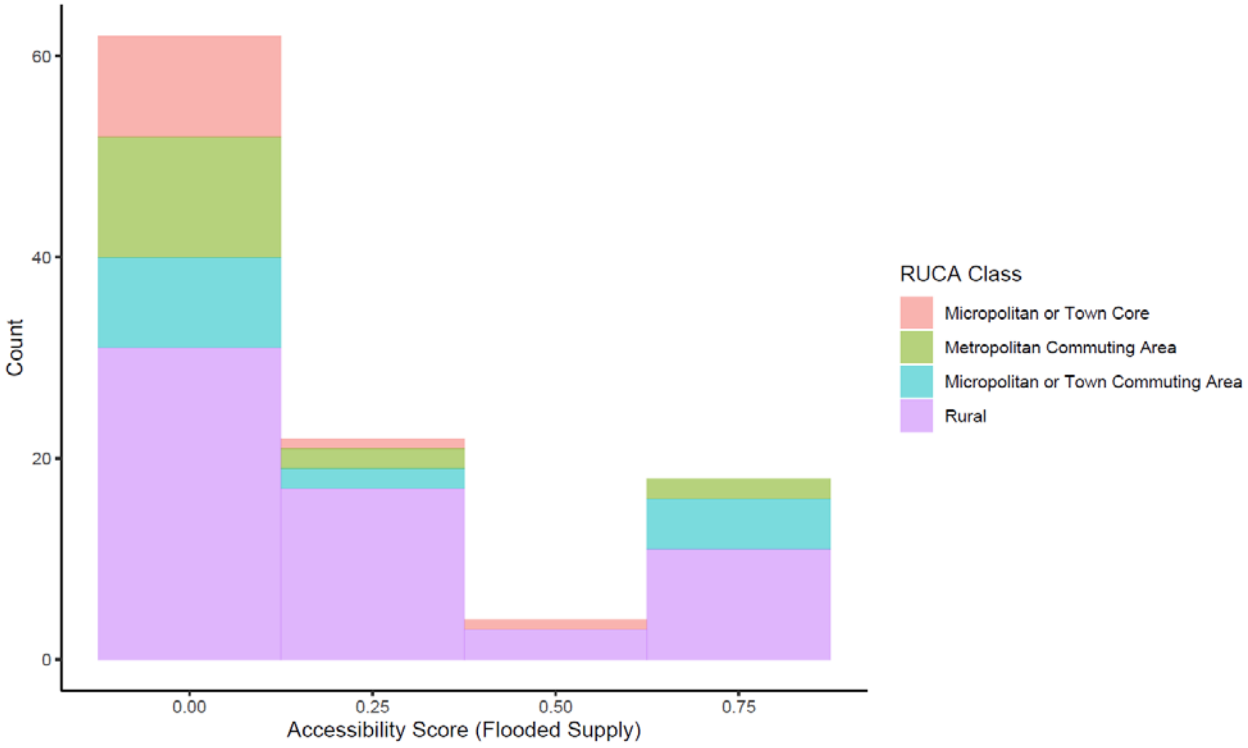

Histogram of accessibility scores of the 106 US Census tracts that remain vaccine deserts when simulating abundant COVID-19 vaccine supply. Census tracts are colored by urbanicity as measured by USDA rural-urban commuting (RUCA) scores.

Supplementary Table 1: Self-reported Percent of U.S. with Pre-existing Conditions from Delphi Survey

Disease	1	2	3	4	5	6	7	8	None	Total N
Self-reported (%)	10.9	5.0	5.3	28.0	15.2	4.1	2.1	7.4	46.8	16,533,319

Diseases are listed as follows: 1. Diabetes, 2. Cancer (other than skin cancer), 3. Heart disease, 4. High blood pressure, 5. Asthma, 6. Chronic lung disease such as COPD or emphysema, 7. Kidney Disease, 8. Autoimmune disorder such as rheumatoid arthritis or Crohn’s disease

Supplementary Table 2: OutbreaksNearMe/SurveyMonkey Respondent* Demographics by Minutes-Willing-to-Travel to COVID-19 Vaccine

	Minutes Willing to Travel to COVID-19 Vaccine					
	**(1,15]**<br>**No. 3,702**	**(15,30]**<br>**No. 10,139**	**(30,45]**<br>**No. 2,171**	**(45,60]**<br>**No. 7,001**	**(60,90]**<br>**No. 4,381**	**Total**<br>**No. 27,394**
Age						
Mean (SD)	48.9 (±16.4)	52.8 (±16.3)	53.1 (±17.1)	53.8 (±16.2)	52.1 (±16.7)	52.4 (±16.5)
Missing	341 (9.2%)	715 (7.1%)	119 (5.5%)	392 (5.6%)	246 (5.6%)	1,813 (6.6%)
Gender						
Female	2,102 (13.4%)	5,997 (38.1%)	1,247 (7.9%)	4,021 (25.6%)	2,362 (15.0%)	15,729 (57.4%)
Male	1,185 (12.5%)	3,307 (34.8%)	782 (8.2%)	2,521 (26.5%)	1,709 (18.0%)	9,504 (34.7%)
Other	74 (21.3%)	120 (34.5%)	23 (6.6%)	67 (19.3%)	64 (18.4%)	348 (1.3%)
Missing	341 (18.8%)	715 (39.4%)	119 (6.6%)	392 (21.6%)	246 (13.6%)	1,813 (6.6%)
Race/Ethnicity						
White	1,981 (10.9%)	6,452 (35.5%)	1,505 (8.3%)	4,967 (27.3%)	3,265 (18.0%)	18,170 (66.3%)
Black	558 (20.4%)	1,181 (43.1%)	201 (7.3%)	537 (19.6%)	265 (9.7%)	2,742 (10.0%)
Hispanic	332 (16.7%)	772 (38.8%)	155 (7.8%)	473 (23.7%)	260 (13.1%)	1,992 (7.3%)
Asian	235 (17.2%)	522 (38.2%)	101 (7.4%)	345 (25.3%)	163 (11.9%)	1,366 (5.0%)
Other	255 (19.5%)	497 (37.9%)	90 (6.9%)	287 (21.9%)	182 (13.9%)	1,311 (4.8%)
Missing	341 (18.8%)	715 (39.4%)	119 (6.6%)	392 (21.6%)	246 (13.6%)	1,813 (6.6%)
Education						
Did not complete high school	119 (18.6%)	240 (37.6%)	53 (8.3%)	130 (20.3%)	97 (15.2%)	639 (2.3%)
High school or G.E.D.	650 (20.3%)	1,341 (41.9%)	212 (6.6%)	682 (21.3%)	312 (9.8%)	3,197 (11.7%)
Some college	737 (15.9%)	1,871 (40.3%)	349 (7.5%)	1,093 (23.6%)	590 (12.7%)	4,640 (16.9%)
Associate's degree	336 (15.7%)	924 (43.1%)	189 (8.8%)	492 (22.9%)	205 (9.6%)	2,146 (7.8%)
College graduate	902 (11.4%)	2,856 (36.1%)	644 (8.1%)	2,111 (26.7%)	1,408 (17.8%)	7,921 (28.9%)
Post graduate degree	617 (8.8%)	2,192 (31.1%)	605 (8.6%)	2,101 (29.9%)	1,523 (21.6%)	7,038 (25.7%)
Missing	341 (18.8%)	715 (39.4%)	119 (6.6%)	392 (21.6%)	246 (13.6%)	1,813 (6.6%)
Household Income						
Under $15,000	337 (23.8%)	564 (39.9%)	105 (7.4%)	263 (18.6%)	144 (10.2%)	1,413 (5.2%)
Between $15,000 and $29,999	405 (20.2%)	871 (43.4%)	137 (6.8%)	403 (20.1%)	191 (9.5%)	2,007 (7.3%)
Between $30,000 and $49,999	520 (17.1%)	1,204 (39.6%)	264 (8.7%)	702 (23.1%)	353 (11.6%)	3,043 (11.1%)
Between $50,000 and $74,999	477 (12.9%)	1,466 (39.6%)	327 (8.8%)	916 (24.8%)	515 (13.9%)	3,701 (13.5%)
Between $75,000 and $99,999	347 (10.9%)	1,202 (37.7%)	288 (9.0%)	897 (28.1%)	458 (14.3%)	3,192 (11.7%)
Between $100,000 and $150,000	393 (9.6%)	1,442 (35.1%)	321 (7.8%)	1,154 (28.1%)	796 (19.4%)	4,106 (15.0%)
Over $150,000	271 (7.3%)	1,066 (28.8%)	310 (8.4%)	1,127 (30.4%)	930 (25.1%)	3,704 (13.5%)
Missing	952 (15.3%)	2,324 (37.3%)	419 (6.7%)	1,539 (24.7%)	994 (16.0%)	6,228 (22.7%)

*Survey results among unvaccinated individuals who responded 'yes' or 'not sure' when asked if they would get the COVID-19 vaccine

Supplementary References:

[1]CDC Diabetes Atlas: https://gis.cdc.gov/grasp/diabetes/DiabetesAtlas.html
[2]Cross-walk files: https://www.huduser.gov/portal/datasets/usps_crosswalk.html
[3]Din, A. & Wilson, R. Crosswalking ZIP Codes to Census Geographies. Cityscape 22, 293–314 (2020).
[4]Thompson, M. G. et al. Interim Estimates of Vaccine Effectiveness of BNT162b2 and mRNA-1273 COVID-19 Vaccines in Preventing SARS-CoV-2 Infection Among Health Care Personnel, First Responders, and Other Essential and Frontline Workers — Eight U.S. Locations, December 2020–March 2021. MMWR. Morb. Mortal. Wkly. Rep. 70, 495–500 (2021).
[5]Liu, Y., Gayle, A. A., Wilder-Smith, A. & Rocklöv, J. The reproductive number of COVID-19 is higher compared to SARS coronavirus. J. Travel Med. 27, 1–4 (2020).
